# Understanding the mechanisms of halotolerance in members of *Pontixanthobacter* and *Allopontixanthobacter* by comparative genome analysis

**DOI:** 10.3389/fmicb.2023.1111472

**Published:** 2023-03-13

**Authors:** Peng Zhou, Yu-Xin Bu, Lin Xu, Xue-Wei Xu, Hong-Bin Shen

**Affiliations:** ^1^Key Laboratory of Marine Ecosystem Dynamics, Ministry of Natural Resources and Second Institute of Oceanography, Ministry of Natural Resources, Hangzhou, China; ^2^College of Life Sciences and Medicine, Zhejiang Sci-Tech University, Hangzhou, China; ^3^School of Oceanography, Shanghai Jiao Tong University, Shanghai, China; ^4^Institute of Image Processing and Pattern Recognition, Shanghai Jiao Tong University, and Key Laboratory of System Control and Information Processing, Ministry of Education of China, Shanghai, China

**Keywords:** halotolerance, co-occurrence, comparative genomics, *Erythrobacteraceae*, adaptation

## Abstract

Halotolerant microorganisms have developed versatile mechanisms for coping with saline stress. With the increasing number of isolated halotolerant strains and their genomes being sequenced, comparative genome analysis would help understand the mechanisms of salt tolerance. Six type strains of *Pontixanthobacter* and *Allopontixanthobacter*, two phylogenetically close genera, were isolated from diverse salty environments and showed different NaCl tolerances, from 3 to 10% (w/v). Based on the co-occurrence greater than 0.8 between halotolerance and open reading frame (ORF) among the six strains, possible explanations for halotolerance were discussed regarding osmolyte, membrane permeability, transportation, intracellular signaling, polysaccharide biosynthesis, and SOS response, which provided hypotheses for further investigations. The strategy of analyzing genome-wide co-occurrence between genetic diversity and physiological characteristics sheds light on how microorganisms adapt to the environment.

## Introduction

Halotolerance is a relative term that refers to the ability to tolerate salt concentrations higher than those necessary for growth, and microorganisms are considered halotolerant if they survive at high salt concentrations but do not require these conditions for growth (Anton, 2014). With advances in technology, halotolerance mechanisms have been investigated using omics approaches. For instance, comparative transcriptomic and physiological analysis revealed that the halotolerant bacterium *Egicoccus halophilus* EGI 80432^T^ increased inorganic ions uptake and accumulated trehalose and glutamate in response to moderate salinity condition, while the high salt condition led to up-regulated transcription of genes required for the synthesis of compatible solutes, such as glutamate, histidine, threonine, proline, and ectoine (Chen et al., 2021). The role of glutamate as a key compatible solute for halotolerance was also reported in a halotolerant strain of *Staphylococcus saprophyticus* based on transcriptome comparison of cells cultivated in media containing different concentrations of NaCl (0, 10, and 20%; Jo et al., 2022). In the exoproteome of the halotolerant bacterium *Tistlia consotensis* grown at high salinity, proteins associated with osmosensing, exclusion of Na^+^ and transport of compatible solutes, such as glycine betaine or proline are abundant (Rubiano-Labrador et al., 2015). Similarly, the proteomic analysis of halotolerant nodule endophytes, *Rahnella aquatilis* strain Ra4 and *Serratia plymuthica* strain Sp2 identified that different trans-membrane ABC transporters (ATP-binding cassettes) were the most represented among the up-regulated proteins in response to salt stress (Novello et al., 2022). Moreover, the proteome comparison of halotolerant bacterium *Staphylococcus aureus* under different osmotic stress conditions revealed the differentially expressed proteins (DEPs) involved in fatty acid synthesis, proline/glycine betaine biosynthesis and transportation, stress tolerance, cell wall biosynthesis, and the TCA cycle, which may contribute to the osmotic stress tolerance of *S. aureus* (Ming et al., 2019). These findings shed light on halotolerance mechanisms. However, halotolerance-related genes may be ignored in transcriptomic and proteomic comparison if there is no significant change in their expression under the experimental conditions.

## Genomic comparisons

Genomic comparisons of closely related halotolerant microorganisms can identify genes conserved among species as well as genes that may give an organism its unique characteristics, which helps to understand the mechanisms of salt tolerance. For example, through comparative genome analysis it was uncovered that the members of *Acidihalobacter* genus contained similar genes for the synthesis and transport of ectoine, as well as genes encoding low affinity potassium pumps. Variations were observed in genes encoding high affinity potassium pumps and proteins involved in the synthesis and/or transport of periplasmic glucans, sucrose, proline, taurine, and glycine betaine (Khaleque et al., 2019). To elucidate salt adaptation strategies in *Nitriliruptoria*, the genomes of five members from group *Nitriliruptoria* were analyzed. The results showed that *Nitriliruptoria* harbor similar synthesis systems of solutes, such as trehalose, glutamine, glutamate, and proline, and on the other hand each member of *Nitriliruptoria* species possesses specific mechanisms, K^+^ influx and efflux, betaine and ectoine synthesis, and compatible solutes transport (Chen et al., 2020). Using whole-genome analysis, the halotolerant strains of *Martelella soudanensis*, NC18^T^ and NC20, were predicted to harbor various halotolerant-associated genes, including K^+^ uptake protein, K^+^ transport system, ectoine transport system, glycine betaine transport system, and glycine betaine uptake protein, indicating that strains NC18^T^ and NC20 might tolerate high salinity through the accumulation of potassium ions, ectoine, glycine betaine (Lee and Kim, 2022). Although these findings help to understand the versatile mechanisms of halotolerance existing in halotolerant microbes, genomic comparisons are usually based on genome-wide searches for homologs of known halotolerance-related genes, such as those involved in K^+^ and Na^+^ influx and efflux and the synthesis and transport of compatible solutes.

The aim of this perspective is to provide new insights into the development of novel hypotheses and promote further studies on the halotolerance mechanisms. Therefore, co-occurrence analysis between halotolerance and open reading frames (ORFs) was performed to provide intuitive information on halotolerance.

## Strains used for analysis

Microorganisms develop abilities that enable them to deal with evolutionary pressure from the environment, such as salinity, temperature, and the power of hydrogen (pH). The phylogenetically closely related strains, which showed similar growth temperature and pH range but different halotolerance, would simplify the analysis. Furthermore, considering the ionic strength of different media may affect the cell growth, the tolerance to NaCl used for co-occurrence analysis should be determined by using same medium. Herein six type strains from two phylogenetically close genera, *Pontixanthobacter* and *Allopontixanthobacter*, were chosen for this study. Because of their close phylogenetic relationship, *Allopontixanthobacter sediminis* and *Allopontixanthobacter confluentis* have been previously classified as *Pontixanthobacter* species (Xu et al., 2020; Liu et al., 2021b), and later were reclassified as *Allopontixanthobacter* species (Xu et al., 2020; Liu et al., 2021a,b). Notably, all the type strains belonging to the two genera were isolated from the Yellow Sea and surrounding areas, but from diverse salty environments, such as *Pontixanthobacter aestiaquae* KCTC 42006^T^ and *Pontixanthobacter rizhaonensis* KCTC 62828^T^ from seawater (Jung et al., 2014; Liu et al., 2021b), *Pontixanthobacter gangjinensis* JCM 17802^T^ and *Pontixanthobacter luteolus* KCTC 12311^T^ from tidal flat (Yoon et al., 2005; Jeong et al., 2013), *Pontixanthobacter aquaemixtae* KCTC 52763^T^ from the junction between ocean and fresh spring (Park et al., 2017), *A. sediminis* KCTC 42453^T^ from lagoon sediments (Kim et al., 2016), and *A. confluentis* KCTC 52259^T^ from water of estuary environment (Park et al., 2017). These strains showed similar optimum NaCl concentrations for growth (1–3%, w/v), but displayed different halotolerances, from 3 to 10% (w/v; Table 1), indicating that these strains adapt to their diverse habitats, including lagoon, junction between ocean and fresh spring, tidal flat, and seawater. The availability of their genomes provides remarkable opportunity to understand their different halotolerances by comparative genome analysis. Here, co-occurrence between halotolerance and the open reading frames (ORFs) was calculated among six strains of *Pontixanthobacter* and *Allopontixanthobacter*, and the ORFs showing high co-occurrence were discussed for possible contribution to halotolerance.

**Table 1:** Strains used for analysis in this study.

Species	Strain	Maximum NaCl (%, w/v)	Optimum NaCl (%, w/v)	Habitat	GenBank accession number
*Pontixanthobacter aestiaquae*	KCTC 42006	10	2–3	Seawater	GCF_009827455.1_ASM982745v1
*Pontixanthobacter gangjinensis*	JCM 17802	9	2	Tidal flat	GCF_009827545.1_ASM982754v1
*Pontixanthobacter luteolus*	KCTC 12311	9	2	Tidal flat	GCF_009828095.1_ASM982809v1
*Pontixanthobacter aquaemixtae*	KCTC 52763	5	2	Junction between ocean and fresh spring	GCF_009827395.1_ASM982739v1
*Allopontixanthobacter sediminis*	KCTC 42453	4	1	Lagoon sediments	GCF_009828115.1_ASM982811v1
*Allopontixanthobacter confluentis*	KCTC 52259	3	1–2	Water of estuary environment	GCF_009827615.1_ASM982761v1

The tolerance of NaCl for all the six strains were investigated based on marine broth (MB). The strain *Pontixanthobacter rizhaonensis* KCTC 62828^T^ was excluded from this study, because it is tested on different medium (Liu et al., 2020, 2021b).

## Clusters highly co-occurred with halotolerance

Open reading frames in the six genomes were predicted and clustered based on similarity using R package micropan (Snipen and Liland, 2015). Analysis of co-occurrence between ORFs and the maximum NaCl concentration tolerated among the six strains was conducted, and 113 clusters of ORFs were identified with co-occurrence greater than 0.8 (Table 2). The co-occurrence for the remaining clusters is listed in Supplementary material, as well as ORFs predicted in the six genomes and the index for clusters and ORFs. ORFs were annotated by searching standard database using protein–protein BLAST.^1^

**Table 2:** Clusters highly co-occurred with halotolerance.

Cluster	Co-occurrence	Annotation
Cluster_111	0.97	TauD/TfdA family dioxygenase
Cluster_229	0.97	Hypothetical protein
Cluster_593	0.97	Metal-dependent hydrolase
Cluster_762	0.97	TonB-dependent receptor
Cluster_1374	0.97	Carbon-nitrogen hydrolase family protein
Cluster_1548	0.97	Sterol desaturase family protein
Cluster_1549	0.97	Endonuclease/exonuclease/phosphatase family protein
Cluster_1706	0.97	Hypothetical protein
Cluster_1740	0.97	ABC transporter ATP-binding protein
Cluster_1899	0.97	VirB4 family type IV secretion/conjugal transfer ATPase
Cluster_2062	0.97	Polysaccharide pyruvyl transferase family protein
Cluster_2063	0.97	Hypothetical protein
Cluster_2065	0.97	EpsG family protein
Cluster_2067	0.97	Glycosyltransferase
Cluster_2069	0.97	Polysaccharide biosynthesis C-terminal domain-containing protein
Cluster_2071	0.97	KpsF/GutQ family sugar-phosphate isomerase
Cluster_2074	0.97	3-Deoxy-manno-octulosonate cytidylyltransferase
Cluster_2076	0.97	3-Deoxy-8-phosphooctulonate synthase
Cluster_2401	0.97	Hypothetical protein
Cluster_2536	0.97	Histone deacetylase
Cluster_2670	0.97	Hypothetical protein
Cluster_2677	0.97	SOS response-associated peptidase family protein
Cluster_614	0.87	Putative quinol monooxygenase
Cluster_1440	0.86	Tail fiber protein
Cluster_1633	0.84	2OG-Fe(II) oxygenase
Cluster_11	0.81	Hypothetical protein
Cluster_12	0.81	DUF885 domain-containing protein
Cluster_59	0.81	PspA/IM30 family protein
Cluster_113	0.81	Hydantoinase B/oxoprolinase family protein
Cluster_115	0.81	DUF969 domain-containing protein
Cluster_116	0.81	DUF979 domain-containing protein
Cluster_117	0.81	DUF2891 domain-containing protein
Cluster_151	0.81	Aldolase/citrate lyase family protein
Cluster_155	0.81	Methyltransferase domain-containing protein
Cluster_166	0.81	Hypothetical protein
Cluster_208	0.81	Trigger factor
Cluster_294	0.81	Enoyl-CoA hydratase-related protein
Cluster_336	0.81	Aspartyl/asparaginyl beta-hydroxylase domain-containing protein
Cluster_395	0.81	Hypothetical protein
Cluster_551	0.81	Hypothetical protein
Cluster_595	0.81	DUF4167 domain-containing protein
Cluster_687	0.81	Amidohydrolase family protein
Cluster_712	0.81	TonB-dependent receptor
Cluster_729	0.81	OmpH family outer membrane protein
Cluster_752	0.81	PilZ domain-containing protein
Cluster_875	0.81	BCCT family transporter
Cluster_879	0.81	Cell division protein ZapA
Cluster_895	0.81	Hypothetical protein
Cluster_983	0.81	GNAT family N-acetyltransferase
Cluster_1081	0.81	DUF805 domain-containing protein
Cluster_1089	0.81	Aminotransferase class IV
Cluster_1090	0.81	Sulfotransferase
Cluster_1132	0.81	Pilus assembly protein TadG-related protein
Cluster_1282	0.81	Hypothetical protein
Cluster_1289	0.81	SDR family oxidoreductase
Cluster_1315	0.81	CinA family protein
Cluster_1328	0.81	Amidohydrolase
Cluster_1340	0.81	Glutathione S-transferase family protein
Cluster_1364	0.81	M2 family metallopeptidase
Cluster_1465	0.81	Hypothetical protein
Cluster_1491	0.81	Hypothetical protein
Cluster_1495	0.81	Serine hydrolase
Cluster_1499	0.81	MarR family transcriptional regulator
Cluster_1565	0.81	Thioesterase family protein
Cluster_1575	0.81	LysR family transcriptional regulator
Cluster_1578	0.81	NAD(P)H-dependent oxidoreductase
Cluster_1663	0.81	Prolyl oligopeptidase family serine peptidase
Cluster_1738	0.81	Lasso peptide biosynthesis B2 protein
Cluster_1739	0.81	Nucleotidyltransferase family protein
Cluster_1741	0.81	Sulfotransferase
Cluster_1742	0.81	Aspartyl beta-hydroxylase
Cluster_1743	0.81	Hypothetical protein
Cluster_1744	0.81	Sulfotransferase domain-containing protein
Cluster_1746	0.81	PqqD family protein
Cluster_1747	0.81	Asparagine synthase-related protein
Cluster_1748	0.81	Glycosyltransferase
Cluster_1838	0.81	DUF3142 domain-containing protein
Cluster_1839	0.81	Hypothetical protein
Cluster_1862	0.81	Hypothetical protein
Cluster_1883	0.81	Isopropylmalate isomerase
Cluster_1896	0.81	Conjugal transfer protein TrbI
Cluster_1901	0.81	VirB3 family type IV secretion system protein
Cluster_1954	0.81	TrbG/VirB9 family P-type conjugative transfer protein
Cluster_1955	0.81	VirB8/TrbF family protein
Cluster_1956	0.81	Type IV secretion system protein
Cluster_2019	0.81	Dipeptidase
Cluster_2022	0.81	Glycerophosphodiester phosphodiesterase family protein
Cluster_2052	0.81	Hypothetical protein
Cluster_2059	0.81	O-antigen ligase family protein
Cluster_2105	0.81	GNAT family N-acetyltransferase
Cluster_2171	0.81	Divalent-cation tolerance protein CutA
Cluster_2209	0.81	DUF2183 domain-containing protein
Cluster_2241	0.81	FKBP-type peptidyl-prolyl cis-trans isomerase
Cluster_2302	0.81	Carbohydrate porin
Cluster_2329	0.81	N-acetyltransferase
Cluster_2345	0.81	NADH:flavin oxidoreductase/NADH oxidase family protein
Cluster_2374	0.81	AI-2E family transporter
Cluster_2384	0.81	Endonuclease III
Cluster_2402	0.81	RNA polymerase sigma factor
Cluster_2408	0.81	GntP family permease
Cluster_2420	0.81	Hypothetical protein
Cluster_2425	0.81	Hypothetical protein
Cluster_2473	0.81	GtrA family protein
Cluster_2474	0.81	Ferritin-like domain-containing protein
Cluster_2475	0.81	Peroxide stress protein YaaA
Cluster_2520	0.81	DsrE family protein
Cluster_2544	0.81	Hypothetical protein
Cluster_2545	0.81	DNA-binding domain-containing protein
Cluster_2546	0.81	Alpha/beta hydrolase
Cluster_2562	0.81	LytTR family DNA-binding domain-containing protein
Cluster_2573	0.81	DUF2306 domain-containing protein
Cluster_2644	0.81	DUF6356 family protein
Cluster_2671	0.81	DUF1295 domain-containing protein

### Osmolyte

The ORFs of Cluster_111 (co-occurrence of 0.97, Table 2) were annotated as TauD/TfdA family dioxygenase. TauD is involved in the utilization of taurine (VanderPloeg et al., 1996), an organic osmolyte involved in cell volume regulation (Harris and Wen, 2012). Taurine is used as an osmoprotectant, such as in *Escherichia coli* at high osmolarity (McLaggan and Epstein, 1991) and in microbial communities from biofilms in metal-rich environment (Mosier et al., 2013). The ORFs of Cluster_111 only exist in three halotolerant strains, suggesting that taurine may be accumulated as an osmoprotectant. Interestingly, halotolerant strains harbor genes involved in various pathways related to glutamate generation. For instance, according to annotation, ORFs of Cluster_113 (co-occurrence of 0.81, Table 2) belong to the hydantoinase B/oxoprolinase family, which includes 5-oxoprolinase, catalyzing the formation of L-glutamate from 5-oxo-L-proline (Niehaus et al., 2017). Besides, ORFs of Cluster_1328 (co-occurrence of 0.81, Table 2) possess similarity to *p*-aminobenzoyl-glutamate (PABA-GLU) hydrolase subunit from *Altererythrobacter insulae* (GenBank Accession Number: RGP41665.1). PABA-GLU is a folate catabolite found in bacteria, and the enzyme PABA-GLU hydrolase breaks down PABA-GLU by cleaving glutamate (Larimer et al., 2014). Additionally, ORFs of Cluster_1747 (co-occurrence of 0.81 Table 2) showed similarity to asparagine synthase from *Salinigranum halophilum* (GenBank Accession Number: WP_136601134.1). Asparagine synthetase catalyzes an ATP-dependent amidotransferase reaction between aspartate and glutamine, which produces asparagine and glutamate (Richards and Kilberg, 2006).

### Permeability

To ensure a physiologically acceptable level of cellular hydration and turgor at high osmolarity, many bacteria accumulate compatible solutes as osmoprotectants (Ziegler et al., 2010). ORFs of Cluster_875 (co-occurrence of 0.81, Table 2) were annotated as proteins of Betaine/Carnitine/Choline Transporter (BCCT) family. The BCCT family includes transporters for carnitine, choline and glycine betaine, and some of which exhibit osmosensory and osmoregulatory properties (Ziegler et al., 2010). Furthermore, the ORFs of Cluster_1740, annotated as ABC transporter ATP-binding proteins, were present only in these three halotolerant strains. The salt-induced ABC transporter Ota from *Methanosarcina mazei* Gö1 acts as a glycine betaine transporter (Schmidt et al., 2007). Another ABC transporter in *Listeria*, OpuC, is shown to be necessary for glycine betaine and choline chloride uptake (Verheul et al., 1997). Compared to the wild type of *S. aureus*, mutating OpuC did reduce their ability to grow under osmotic stress (10% NaCl; Kiran et al., 2009). The function of ORFs of Cluster_1740 and their contribution to halotolerance can be further characterized. Additionally, previous studies have shown that water permeability is clearly affected by the number of double bonds in the fatty acid conjugates of lipids, the higher the degree of unsaturation, the greater the water permeability (Graziani and Livne, 1972), and sterol type is one of the determining factors in the permeability of membranes to small solutes (Frallicciardi et al., 2022). The genomes of three halotolerant strains contain ORFs of Cluster_1548, annotated as sterol desaturase family proteins, indicating that sterols might be used to change permeability.

### Cell signaling

Cluster_1549 also consists of three ORFs present in the three halotolerant strains, which showed similarity to the domain superfamily found in a large number of proteins including magnesium dependent endonucleases and phosphatases involved in intracellular signaling (Dlakic, 2000). Its role in the regulation of gene expression, such as triggering the salt-stress response, is worth of further study.

### Polysaccharide

It has been reported that extracellular polysaccharides (EPS) may influence the salt tolerance of certain rhizobial strains (Samir and Kanak, 1997) and the lipopolysaccharide pattern could alter according to different salinities in a salt-tolerant strain of *Mesorhizobium cicero* (Soussi et al., 2001). All three halotolerant strains harbor ORFs annotated with polysaccharide/lipopolysaccharide biosynthesis (Cluster_2062, 2065, 2067, 2069, 2071, 2074, and 2076 in Table 2), such as 3-deoxy-d-manno-octulosonate cytidylyltransferase, a key enzyme in the biosynthesis of lipopolysaccharide (LPS) in Gram-negative organisms (Yi et al., 2011). Furthermore, ORFs of Cluster_2473 (co-occurrence as 0.81 Table 2) were annotated to encode proteins of the GtrA family, whose members are often involved in the synthesis of cell surface polysaccharides (Kolly et al., 2015).

### DNA repair

Open reading frames of Cluster_2677 are annotated encoding SOS response-associated peptidase family protein. The bacterial SOS response induced under stress conditions is recruited to DNA repair and adaptive mutagenesis (Shinagawa, 1996; Aravind et al., 2013). Hence, ORFs of Cluster_2677 could be further investigated for its importance to halotolerance.

## Discussion

Salinity is one of the most important environmental factors for aquatic microorganisms and varies among habitats. Therefore, halotolerant microorganisms have developed versatile strategies to cope with saline stress. Based on the findings of co-occurrence analysis, possible explanations for mechanisms resulting in different salt tolerances among six strains are discussed above, which provided hypotheses for further investigations. Moreover, among the highly co-occurred clusters, there are several uncharacterized or hypothetical proteins (Table 2), which may contribute to halotolerance. It should be noted that the genes related to resistance to salts other than sodium chloride could also be discovered by co-occurrence analysis, since various salts co-exist in high ionic environments. For instance, ORFs of Cluster_2171 (co-occurrence as 0.81, Table 2) were annotated as divalent-cation tolerance protein CutA, which is required for copper tolerance in *E. coli* and affects tolerance levels to zinc, nickel, cobalt, and cadmium salts (Fong et al., 1995). This study sheds light on the mechanisms through which microorganisms cope with environmental stress. With the increasing number of isolated halotolerant strains and their genomes being sequenced, analyzing genome-wide co-occurrence between genetic diversity and physiological characteristics would expand the knowledge of the salinity adaptation strategies and provide comprehensive information on how microorganisms adapt to the environment, together with findings at the transcriptomic and proteomic levels.

## Data availability statement

Publicly available datasets were analyzed in this study. This data can be found here: https://www.ncbi.nlm.nih.gov. Accession Numbers are as follows: GCF_009827455.1_ASM982745v1, GCF_009827545.1_ASM982754v1, GCF_009828095.1_ASM982809v1, GCF_009827395.1_ASM982739v1, GCF_009828115.1_ASM982811v1, and GCF_009827615.1_ASM982761v1.

## Author contributions

PZ contributed to study concept and design and performed data acquisition, analysis and visualization, and interpretation of results. PZ and Y-XB drafted the manuscript. LX, X-WX, and H-BS revised the manuscript. All authors contributed to the article and approved the submitted version.

## Funding

This work was supported by grants from the National Science and Technology Fundamental Resources Investigation Program of China (2021FY100900), the Scientific Research Fund of the Second Institute of Oceanography, MNR (No. JZ1901 and JB2003), Natural Science Foundation of China (32000001), and the Oceanic Interdisciplinary Program of Shanghai Jiao Tong University (SL2022ZD108).

## Conflict of interest

The authors declare that the research was conducted in the absence of any commercial or financial relationships that could be construed as a potential conflict of interest.

## Publisher’s note

All claims expressed in this article are solely those of the authors and do not necessarily represent those of their affiliated organizations, or those of the publisher, the editors and the reviewers. Any product that may be evaluated in this article, or claim that may be made by its manufacturer, is not guaranteed or endorsed by the publisher.

## Supplementary material

The Supplementary material for this article can be found online at: https://www.frontiersin.org/articles/10.3389/fmicb.2023.1111472/full#supplementary-material

## Footnotes

^1^ https://blast.ncbi.nlm.nih.gov

## References

[ref1] Anton J. (2014). “Halotolerance” in Encyclopedia of Astrobiology. eds. Amils R. Gargaud M. Quintanilla J. C. Cleaves H. J. Irvine W. M. Pinti D.. (Berlin, Heidelberg: Springer Berlin Heidelberg), 1–2.

[ref2] Aravind L. Anand S. Iyer L. M. (2013). Novel autoproteolytic and DNA-damage sensing components in the bacterial SOS response and oxidized methylcytosine-induced eukaryotic DNA demethylation systems. Biol. Direct 8:20. doi: 10.1186/1745-6150-8-20, PMID: 23945014 PMC3765255

[ref3] Chen D. D. Fang B. Z. Manzoor A. Liu Y. H. Li L. Mohamad O. A. A.. (2021). Revealing the salinity adaptation mechanism in halotolerant bacterium *Egicoccus halophilus* EGI 80432(T) by physiological analysis and comparative transcriptomics. Appl. Microbiol. Biotechnol. 105, 2497–2511. doi: 10.1007/s00253-021-11190-5, PMID: 33625547

[ref4] Chen D. D. Tian Y. Jiao J. Y. Zhang X. T. Zhang Y. G. Dong Z. Y.. (2020). Comparative genomics analysis of Nitriliruptoria reveals the genomic differences and salt adaptation strategies. Extremophiles 24, 249–264. doi: 10.1007/s00792-019-01150-3, PMID: 31820112

[ref5] Dlakic M. (2000). Functionally unrelated signalling proteins contain a fold similar to Mg^2+^−dependent endonucleases. Trends Biochem. Sci. 25, 272–273. doi: 10.1016/s0968-0004(00)01582-6, PMID: 10838565

[ref6] Fong S.-T. Camakaris J. Lee B. T. O. (1995). Molecular genetics of a chromosomal locus involved in copper tolerance in *Escherichia coli* K-12. Mol. Microbiol. 15, 1127–1137. doi: 10.1111/j.1365-2958.1995.tb02286.x, PMID: 7623666

[ref7] Frallicciardi J. Melcr J. Siginou P. Marrink S. J. Poolman B. (2022). Membrane thickness, lipid phase and sterol type are determining factors in the permeability of membranes to small solutes. Nat. Commun. 13:1605. doi: 10.1038/s41467-022-29272-x, PMID: 35338137 PMC8956743

[ref8] Graziani Y. Livne A. (1972). Water permeability of bilayer lipid membranes: sterol-lipid interaction. J. Membr. Biol. 7, 275–284. doi: 10.1007/BF01867920, PMID: 24177511

[ref9] Harris R. Wen S. (2012). Review: Taurine: A “very essential” amino acid. Mol Vis. 18, 2673–2686. 23170060 PMC3501277

[ref10] Jeong S. H. Jin H. M. Lee H. J. Jeon C. O. (2013). *Altererythrobacter gangjinensis* sp. nov., a marine bacterium isolated from a tidal flat. Int. J. Syst. Evol. Microbiol. 63, 971–976. doi: 10.1099/ijs.0.039024-0, PMID: 22685101

[ref11] Jo E. Hwang S. Cha J. (2022). Transcriptome analysis of Halotolerant *Staphylococcus saprophyticus* isolated from Korean fermented shrimp. Foods 11:524. doi: 10.3390/foods11040524, PMID: 35206000 PMC8870806

[ref12] Jung Y. T. Park S. Lee J. S. Yoon J. H. (2014). *Altererythrobacter aestiaquae* sp. nov., isolated from seawater. Int. J. Syst. Evol. Microbiol. 64, 3943–3949. doi: 10.1099/ijs.0.066639-0, PMID: 25201916

[ref13] Khaleque H. N. Gonzalez C. Shafique R. Kaksonen A. H. Holmes D. S. Watkin E. L. J. (2019). Uncovering the mechanisms of Halotolerance in the extremely acidophilic members of the *Acidihalobacter* genus through comparative genome analysis. Front. Microbiol. 10:155. doi: 10.3389/fmicb.2019.00155, PMID: 30853944 PMC6396713

[ref14] Kim J. H. Yoon J. H. Kim W. (2016). *Altererythrobacter sediminis* sp. nov., isolated from lagoon sediments. Int. J. Syst. Evol. Microbiol. 66, 5424–5429. doi: 10.1099/ijsem.0.001535, PMID: 27692040

[ref15] Kiran M. D. Akiyoshi D. E. Giacometti A. Cirioni O. Scalise G. Balaban N. (2009). OpuC—an ABC transporter that is associated with *Staphylococcus aureus* pathogenesis. Int. J. Artif. Organs 32, 600–610. doi: 10.1177/039139880903200909, PMID: 19856269

[ref16] Kolly G. S. Mukherjee R. Kilacskova E. Abriata L. A. Raccaud M. Blasko J.. (2015). GtrA protein Rv3789 is required for Arabinosylation of Arabinogalactan in *Mycobacterium tuberculosis*. J. Bacteriol. 197, 3686–3697. doi: 10.1128/JB.00628-15, PMID: 26369580 PMC4626892

[ref17] Larimer C. M. Slavnic D. Pitstick L. D. Green J. M. (2014). Comparison of substrate specificity of *Escherichia coli* p-Aminobenzoyl-glutamate hydrolase with *Pseudomonas* Carboxypeptidase G. Adv. Enzyme Res. 2, 39–48. doi: 10.4236/aer.2014.21004, PMID: 27795973 PMC5082436

[ref18] Lee J. Y. Kim D. H. (2022). Genomic analysis of Halotolerant bacterial strains *Martelella soudanensis* NC18(T) and NC20. J. Microbiol. Biotechnol. 32, 1427–1434. doi: 10.4014/jmb.2208.08011, PMID: 36330756 PMC9720073

[ref19] Liu A. Xue Q. J. Li S. G. Zhang Y. J. (2021a). Corrigendum: *Pontixanthobacter rizhaonensis* sp. nov., a marine bacterium isolated from surface seawater of the Yellow Sea, and the proposal of *Pseudopontixanthobacter* gen. Nov., *Pseudopontixanthobacter confluentis* comb. nov. and *Pseudopontixanthobacter sediminis* comb. nov. Int. J. Syst. Evol. Microbiol. 71:004931. doi: 10.1099/ijsem.0.004931, PMID: 33886448

[ref20] Liu A. Xue Q. J. Li S. G. Zhang Y. J. (2021b). *Pontixanthobacter rizhaonensis* sp. nov., a marine bacterium isolated from surface seawater of the Yellow Sea, and proposal of *Pseudopontixanthobacter* gen. Nov., *Pseudopontixanthobacter confluentis* comb. nov. and *Pseudopontixanthobacter sediminis* comb. nov. Int. J. Syst. Evol. Microbiol. 71:004780. doi: 10.1099/ijsem.0.004780 33886448

[ref21] Liu A. Zhang Y. J. Xue Q. J. Wang H. Yang Y. Y. Du F.. (2020). *Litorilituus lipolyticus* sp. nov., isolated from intertidal sand of the Yellow Sea in China, and emended description of *Colwellia asteriadis*. Antonie Van Leeuwenhoek 113, 449–458. doi: 10.1007/s10482-019-01355-8, PMID: 31701358

[ref22] McLaggan D. Epstein W. (1991). *Escherichia coli* accumulates the eukaryotic osmolyte taurine at high osmolarity. FEMS Microbiol. Lett. 81, 209–213. doi: 10.1016/0378-1097(91)90304-s, PMID: 1884995

[ref23] Ming T. Geng L. Feng Y. Lu C. Zhou J. Li Y.. (2019). iTRAQ-based quantitative proteomic profiling of *Staphylococcus aureus* under different osmotic stress conditions. Front. Microbiol. 10:1082. doi: 10.3389/fmicb.2019.01082, PMID: 31191466 PMC6549500

[ref24] Mosier A. C. Justice N. B. Bowen B. P. Baran R. Thomas B. C. Northen T. R.. (2013). Metabolites associated with adaptation of microorganisms to an acidophilic, metal-rich environment identified by stable-isotope-enabled metabolomics. MBio 4, e00484–e00412. doi: 10.1128/mBio.00484-12 23481603 PMC3604775

[ref25] Niehaus T. D. Elbadawi-Sidhu M. de Crecy-Lagard V. Fiehn O. Hanson A. D. (2017). Discovery of a widespread prokaryotic 5-oxoprolinase that was hiding in plain sight. J. Biol. Chem. 292, 16360–16367. doi: 10.1074/jbc.M117.805028, PMID: 28830929 PMC5625064

[ref26] Novello G. Gamalero E. Massa N. Cesaro P. Lingua G. Todeschini V.. (2022). Proteome and physiological characterization of Halotolerant nodule Endophytes: the case of *Rahnella aquatilis* and *Serratia plymuthica*. Microorganisms 10:890. doi: 10.3390/microorganisms10050890, PMID: 35630335 PMC9143289

[ref27] Park S. Jung Y. T. Choi S. J. Yoon J. H. (2017). *Altererythrobacter aquaemixtae* sp. nov., isolated from the junction between the ocean and a freshwater spring. Int. J. Syst. Evol. Microbiol. 67, 3446–3451. doi: 10.1099/ijsem.0.002136, PMID: 28840800

[ref28] Richards N. G. Kilberg M. S. (2006). Asparagine synthetase chemotherapy. Annu. Rev. Biochem. 75, 629–654. doi: 10.1146/annurev.biochem.75.103004.142520, PMID: 16756505 PMC3587692

[ref29] Rubiano-Labrador C. Bland C. Miotello G. Armengaud J. Baena S. (2015). Salt stress induced changes in the exoproteome of the Halotolerant bacterium *Tistlia consotensis* deciphered by Proteogenomics. PLoS One 10:e0135065. doi: 10.1371/journal.pone.0135065, PMID: 26287734 PMC4545795

[ref30] Samir K. M. Kanak R. S. (1997). The correlation between salt tolerance and extracellular Polysaccharid. Microbes Environ. 12, 9–13.

[ref31] Schmidt S. Pfluger K. Kogl S. Spanheimer R. Muller V. (2007). The salt-induced ABC transporter Ota of the methanogenic archaeon *Methanosarcina mazei* Go1 is a glycine betaine transporter. FEMS Microbiol. Lett. 277, 44–49. doi: 10.1111/j.1574-6968.2007.00938.x, PMID: 17986083

[ref32] Shinagawa H. (1996). SOS response as an adaptive response to DNA damage in prokaryotes. EXS 77, 221–235. doi: 10.1007/978-3-0348-9088-5_14, PMID: 8856977

[ref33] Snipen L. Liland K. H. (2015). Micropan: an R-package for microbial pan-genomics. BMC Bioinf. 16:79. doi: 10.1186/s12859-015-0517-0, PMID: 25888166 PMC4375852

[ref34] Soussi M. Santamaria M. Ocana A. Lluch C. (2001). Effects of salinity on protein and lipopolysaccharide pattern in a salt-tolerant strain of *Mesorhizobium ciceri*. J. Appl. Microbiol. 90, 476–481. doi: 10.1046/j.1365-2672.2001.01269.x, PMID: 11298245

[ref35] VanderPloeg J. R. Weiss M. A. Saller E. Nashimoto H. Saito N. Kertesz M. A.. (1996). Identification of sulfate starvation-regulated genes in *Escherichia coli*: a gene cluster involved in the utilization of taurine as a sulfur source. J. Bacteriol. 178, 5438–5446. doi: 10.1128/jb.178.18.5438-5446.1996, PMID: 8808933 PMC178364

[ref36] Verheul A. Glaasker E. Poolman B. Abee T. (1997). Betaine and L-carnitine transport by *Listeria monocytogenes* Scott a in response to osmotic signals. J. Bacteriol. 179, 6979–6985. doi: 10.1128/jb.179.22.6979-6985.1997, PMID: 9371443 PMC179637

[ref37] Xu L. Sun C. Fang C. Oren A. Xu X. W. (2020). Genomic-based taxonomic classification of the family *Erythrobacteraceae*. Int. J. Syst. Evol. Microbiol. 70, 4470–4495. doi: 10.1099/ijsem.0.004293, PMID: 32726199 PMC7660246

[ref38] Yi L. Velasquez M. S. Holler T. P. Woodard R. W. (2011). A simple assay for 3-deoxy-d-manno-octulosonate cytidylyltransferase and its use as a pathway screen. Anal. Biochem. 416, 152–158. doi: 10.1016/j.ab.2011.05.022, PMID: 21669179 PMC3380378

[ref39] Yoon J. H. Kang K. H. Yeo S. H. Oh T. K. (2005). *Erythrobacter luteolus* sp. nov., isolated from a tidal flat of the Yellow Sea in Korea. Int. J. Syst. Evol. Microbiol. 55, 1167–1170. doi: 10.1099/ijs.0.63522-0, PMID: 15879250

[ref40] Ziegler C. Bremer E. Kramer R. (2010). The BCCT family of carriers: from physiology to crystal structure. Mol. Microbiol. 78, 13–34. doi: 10.1111/j.1365-2958.2010.07332.x, PMID: 20923416

